# Analysis of Fluoride Contamination in Sheep Serum and in Water in the North East of Tunisia Ore Deposits

**DOI:** 10.3389/ftox.2021.643664

**Published:** 2021-07-19

**Authors:** Rim Hadiji, Iheb Bannouri, Saida Zelfani, Aymen Mamlouk, Hela Jaballah, Arij Jemmal, Nadia Sdouga, Samir Ben Youssef

**Affiliations:** ^1^National School of Veterinary Medicine, Sidi Thabet, Manouba University, Ariana, Tunisia; ^2^Faculty of Medicine, University El Manar of Tunis, Tunis, Tunisia; ^3^Department of Animal Production, Zaghouan, Tunisia

**Keywords:** fluor, serology, Tunisia, sheep, water, analysis

## Abstract

The north east of Tunisia ore deposits (Hammam Zriba) contain a large amount of fluorides constituting a risk of environmental pollution as well as risks to human and animal health. The aim of our work was to assess the levels of fluorides in the water and blood of sheep in the east of Tunisia region. This analysis study included 78 water samples and 60 blood samples taken during 3 days in the year 2019, from a sheep herd at Hammam Zriba delegation. The determination of fluorine concentration in the samples was carried out by potentiometry with a selective electrode type ISE combined Fluoride perfectION™. High Fluoride concentrations were found at a mean of 1.62 ± 1.18, 1.45 ± 0.98, and 2.65 ± 1.61 mg/l, respectively, in running, deep, and stagnant waters. In addition, 83.3% of the animals reared within 400 m of the study area had elevated fluoride levels in the blood exceeding the usual values (fluoremia> 0.15 mg/l). The concentration of fluoride in the blood of the animals decreases with increasing distance from the mine.

## Introduction

Fluoride is the 13th most abundant element in the earth's crust. It is essential for human and animal health. Animals are exposed to fluoride ions particulary by consuming water rich in fluorine. Ingesting small amounts of fluoride in water is generally beneficial for teeth and bones, especially in young animals (World Health Organization, 2017).

The permissible limit for fluorides in drinking water is 1.5 mg/l (World Health Organization, 2017). However, long-term ingestion of large amounts can cause serious dental and bone problems in animals.

Controlling the quality of drinking water is therefore essential to prevent animal fluorosis, which results in dental lesions (brown enamel stains + chalky appearance and irregularity of the molar table) and bone lesions (exostoses) affecting the mandible, ribs, metacarpus, and metatarsus (National Research Council, 2006). Fluorosis is also responsible for a decline in zootechnical performance which explains the economic losses and even the difficulty to develop sheep farming in Tunisia.

Fluorides occur naturally in the environment. Because of its high reactivity, fluorine is never present in its elemental state in nature. It is always found in combined form with other organic or mineral elements that cause toxicity problems affecting fauna, flora, and the environment.

Fluoride pollution comes from human activities such as agriculture, urbanization, industrialization, and mining (Facchineli et al., 2001; Djebbi et al., 2017). Indeed, the tailings of mining waste landfills coming from smelting and extraction procedures, including process fluids from factories, the remaining fluids after the extraction of minerals, metals, fuels, or coal contribute to the environmental contamination (Djebbi et al., 2017).

The impact of wind on atmospheric particles and dry or wet deposits are primary pathways for atmospheric dispersion and transport of this pollutant, which subsequently affects water, fauna, and flora.

Fluorides can also be transported by leaching from precipitation and contaminate streams and groundwater. Wind, by comparison, rapidly transports fluoridated airborne dust over long distances. Thus, the main risk of airborne fluoride dust emissions from abandoned mining areas is the presence of unconfined fluorite tailings ponds containing high fluoride loadings that are either wind-borne or transported by water runoff or leached to groundwater (Adjagodo and Agassounon, 2017; Djebbi et al., 2017).

Few studies have evaluated the quantities of fluorides, emitted by the mining waste, in the waters and in living animals near the fluorite mine located in the east of Tunisia (Botha et al., 1993). In a Mediterranean climate, with little rainfall and low ground cover, fluoride dust from mine tailings can specifically affect the environment surrounding the mine.

The objective of our work was to study the level of fluoride contamination in the deposit region of Hammam zriba by determining the fluoride levels in the water and blood of sheep living in the study area.

## Methods

### Collection of Samples

**Figure 1:**
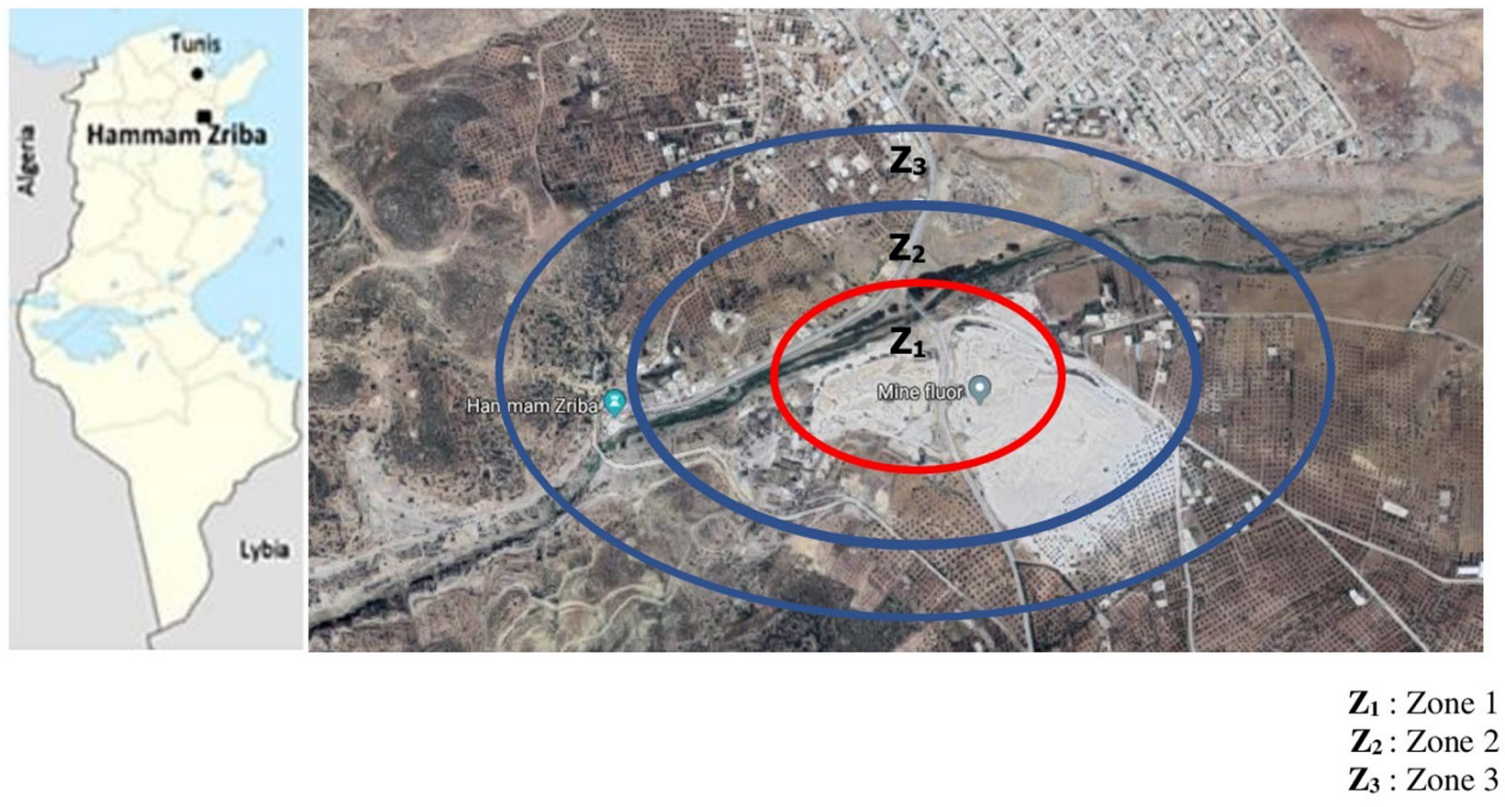
Location of study area and sampling points.

Our work was carried out on 2 types of samples: water and sheep's blood. In the study area, sampling was performed over three points. A reference site was used in order to determine the difference in fluoride accumulation in the environment of the mines (Figure 1).

All the samples were taken in Novembre 2019 in order to assess the level of fluoride contamination in water and blood animals. The concentration of fluoride ions in the different water and blood samples was determined by potentiometry using a fluoride ion selective electrode of the ISE type combined Fluoride perfectION™.

The water samples are divided into three groups according to the distance from the mine:

- E1 [Mine-400 m]
- E2 [400 m−1 Km]
- E3 [>2 Km]

The water sampling concerned surface water, groundwater, and drinking water in the in the ore deposit region of Hammam Zriba. These samples are distributed to cover most of the region and are selected locally based on the number of animals consuming the water. The water samples are then stored in polyethylene sterile bottles to avoid contamination.

Sheep's blood (10 ml) was collected from apparently healthy sheeps living in Hammam Zriba region. All the blood samples were taken during 3 consecutive days in the year 2019 to make sure that the animals did not move between the zones. After the skin was cleaned with 70% ethanol, blood samples were taken from the jugular vein and collected in heparinized tubes. All samples were taken from adult animals between 4 and 8 years. The blood was then centrifuged at 3,000 rpm for 10 min within 4 h of collection. Plasma was used for the assay. This plasma was then recovered in test tubes and stored at +4°C.

We divided the animals into three groups: S1, S2, and S3 according to the location of their farms in regard to the mine.

- S1:between 0 and 400 m from the mine (18 sheep)
- S2:between 400 and 1,000 m from the mine (18 sheep)
- S3: ≥ at 2,000 m from the mine (24 sheep)

In this study, we defined by fluoremia the levels of fluoride concentration in sheep serum and by fluorisis the chronic or long-term poisoning due to repeated ingestion of foods or water containing high levels of fluoride (World Health Organization, 2017).

### Ethics

The ethics approval or specific consent procedures are not required for the study: “Analysis of fluoride concentrations in sheep serum and in water in the north east of Tunisia ore deposits.”

There was no animal experimentation in this study. Only blood samples were taken as part of the animal health assessment.

Consent was obtained from owners for their animal's participation in this study.

The animal study was reviewed and approved by a veterinary practitioner in accordance with good practices related to the sampling.

### Reagents and Laboratory Ware

The analysis method is developed and validated at the laboratory of Pharmacy and Toxicology at the National School of Veterinary Medicine-Tunisia. It is used to measure the concentration of fluorides in solutions where fluoride concentrations are higher than 0.02 mg/l (detection limit). Therefore, from a sodium fluoride main solution (100 mg/l), five standard solutions with concentrations ranging from 10^−1^ to 10^−6^mg/l have been prepared.

The concentration of fluoride ions in the different water and blood samples was determined by potentiometry using a fluoride ions selective electrode of the ISE type combined Fluoride perfectION™.

The determination of fluoride ions in the samples was done after the addition of an equal volume of Total Ionic Strength Adjustment Buffer with the following composition: Glacial acetic acid (57 ml); Sodium chloride (58 g); Sodium citrate (0.3 g) and bi-distillated water (500 ml). The solution is adjusted to pH 5–5.5 with 5 M sodium hydroxide solution. This acidic solution is added to the buffer by successive additions with a graduated pipette. The pH control was done progressively with a calibrated pH meter. It is cooled and then made up to 1 liter with bi-distillated water.

In order to obtain the most significant results possible, we have procured the purest fluoride free reactants possible and used doubly distilled water for all handling.

Each sample was tested twice. A coefficient of variation ≤ 2% was used to validate the measurement.

### Statistical Analysis

The data were analyzed using SPSS (version 22.0). All data were expressed as mean ± SD and the level of significance was determined at *p* < 0.05.

## Results

In total, 78 water samples and 60 blood samples were taken.

### Variation of Fluoride Concentration in the Water

In the 78 water samples, we noted a positive correlation between the mean of fluoride concentration (1.92 ± 1.38 mg/l) and the mean of pH water (9.39 ± 0.27) with a coefficient *r* = 0.79, and *p* < 0.001.

Each group consisted of 36 samples. The mean fluoride concentration was, respectively, 2.94 ± 1.55 mg/l in first group E1, 1.79 ± 0.96 mg/l in the second group E2, and 1.03 ± 0.83 mg/l in the third group E3. The difference between three groups was statistically significant and *p* < 0.001.

**Figure 2:**
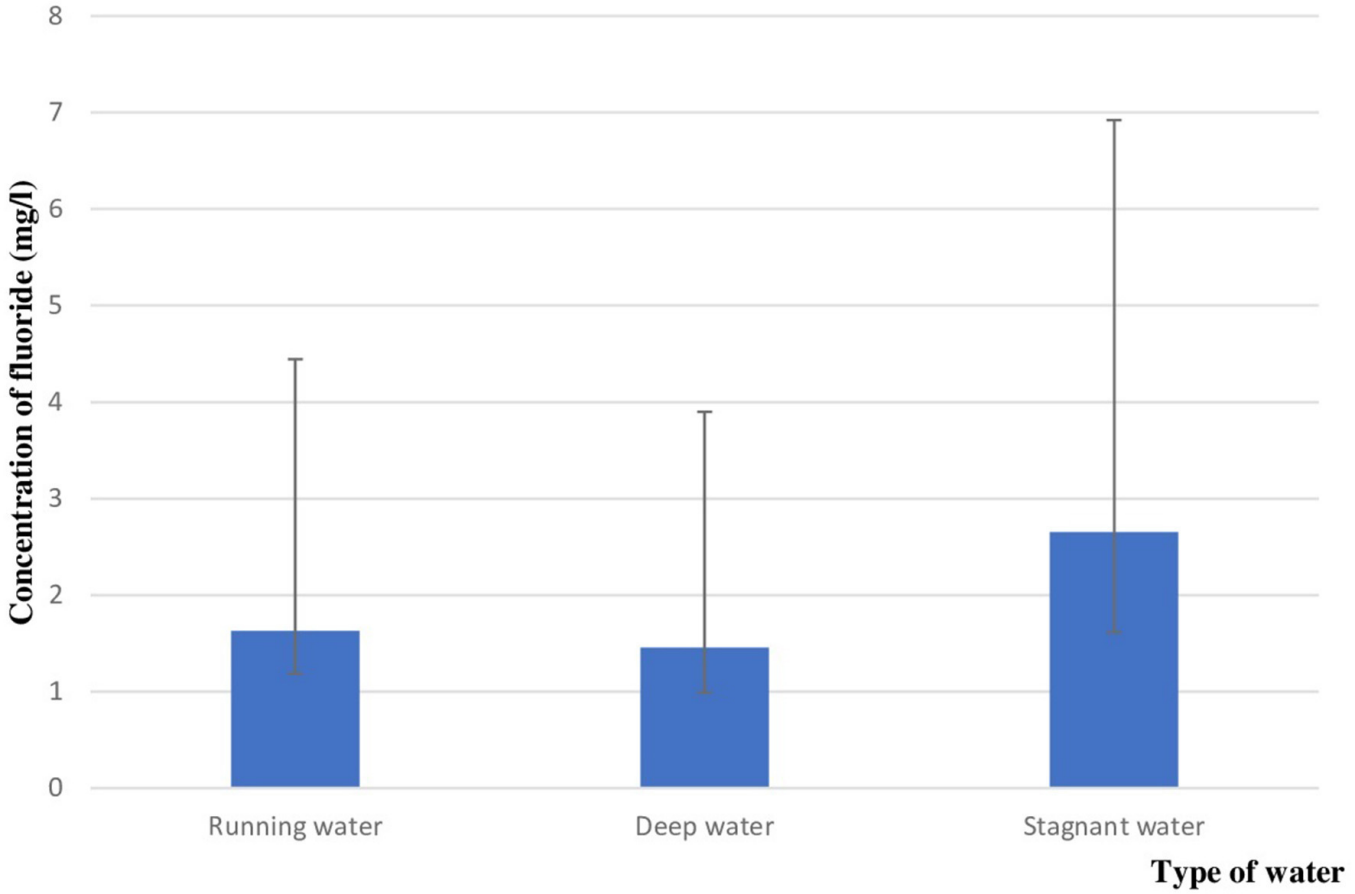
Fluoride concentration depending on the type of water.

Comparison of the fluoride concentration according to the type of water demonstrated an average of fluoride concentration equal to: 1.62 ± 1.18 mg/l in the running water (32 samples), 1.45 ± 0.98 mg/l in the deep water (20 samples), and 2.65 ± 1.61 mg/l in the stagnant water (26 samples) as shown in the Figure 2.

The difference between this three groups was statistically significant (*p* = 0.003).

### Variation of Fluoremia

#### Effect of Age on Fluoremia

Five animals (71.4%) aged more than 4 years had high fluoremia (fluoremia> 0.15 mg/l).

The mean of fluoremia for this age category was 0.36 ± 0.2 mg/l.

Thirteen sheeps (24.5%) aged 4 years had fluoremia exceeding the threshold with an average of 0.15 ± 0.11 mg/l for this category. The difference in fluoremia between these two groups was statistically significant (*p* = 0.011).

**Figure 3:**
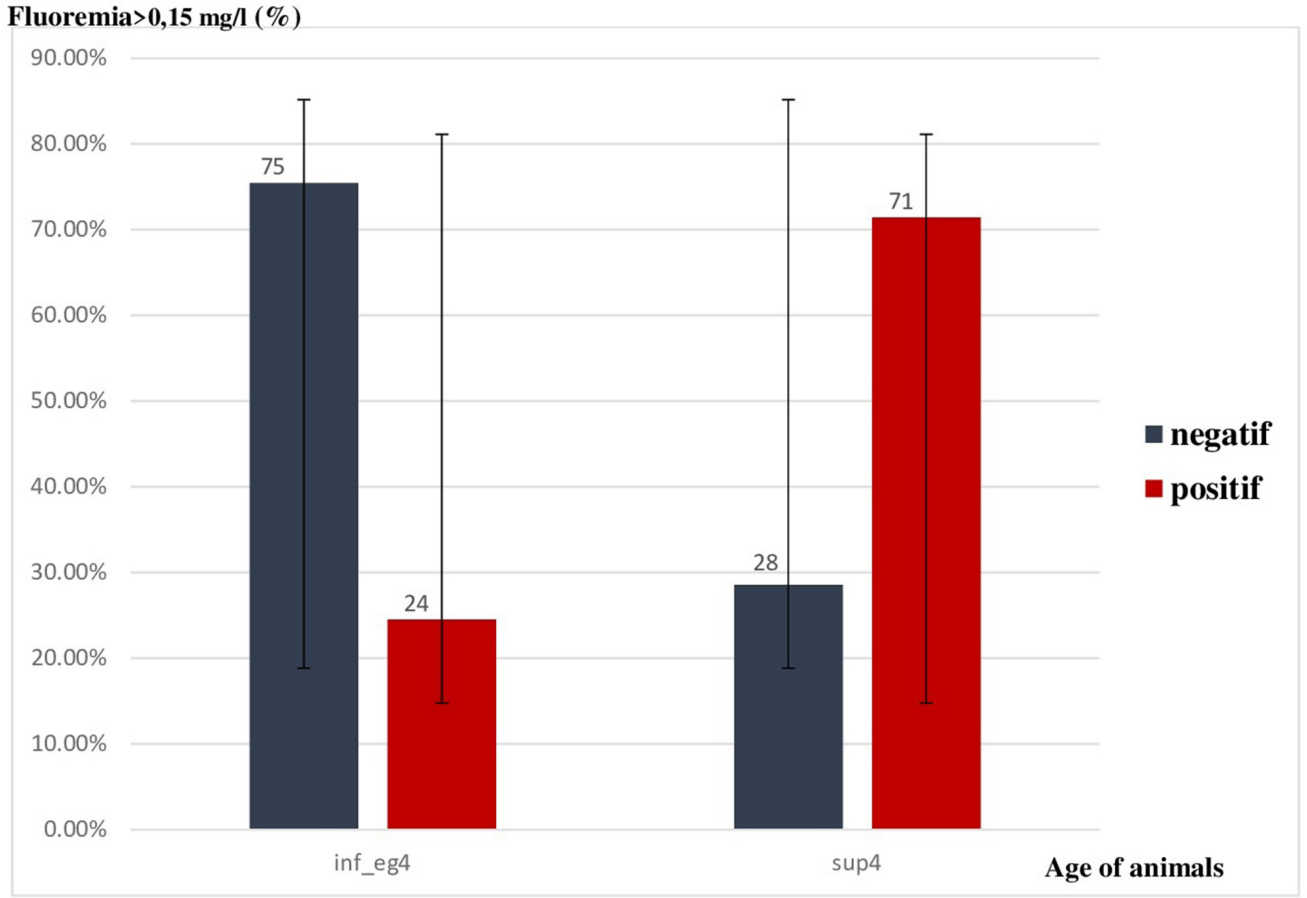
Variation of fluoremia by age of animals.

Variation of fluoremia by âge of sheeps was represented in the Figure 3.

#### Effect of Gender on Fluoremia

In sheep serum, 18 females (32.1%) had elevated fluoremia above cutoff; In contrast, 4 males (100%) had normal fluoremia. But there was no statistically significant difference (*p* = 0.17).

#### Effect of Distance From the Mine on Fluoremia

Fifteen sheep (83.3%) whose breeding was located in the range 0–400 m were positive with elevated levels of fluoride in the blood exceeding the threshold set (fluoremia> 0.15 mg/l) as shown in Table 1.

**Table 1:** Fluoride concentration as a function of distance.

		**Fluoremia**	
		** <0.15 mg/l**	**>0.15 mg/l**
Distance (meters)	S1 *n* (%) D-[0–400]	3 (16.7)	15 (83.3)
	S2 *n* (%) D-[400–1,000]	17 (94.4)	1 (5.6)
	S3 *n* (%) D-≥2,000	22 (91.7)	2 (8.3)

The difference observed with other farms located further away from the mine was statistically significant (*p* < 0.001).

**Figure 4:**
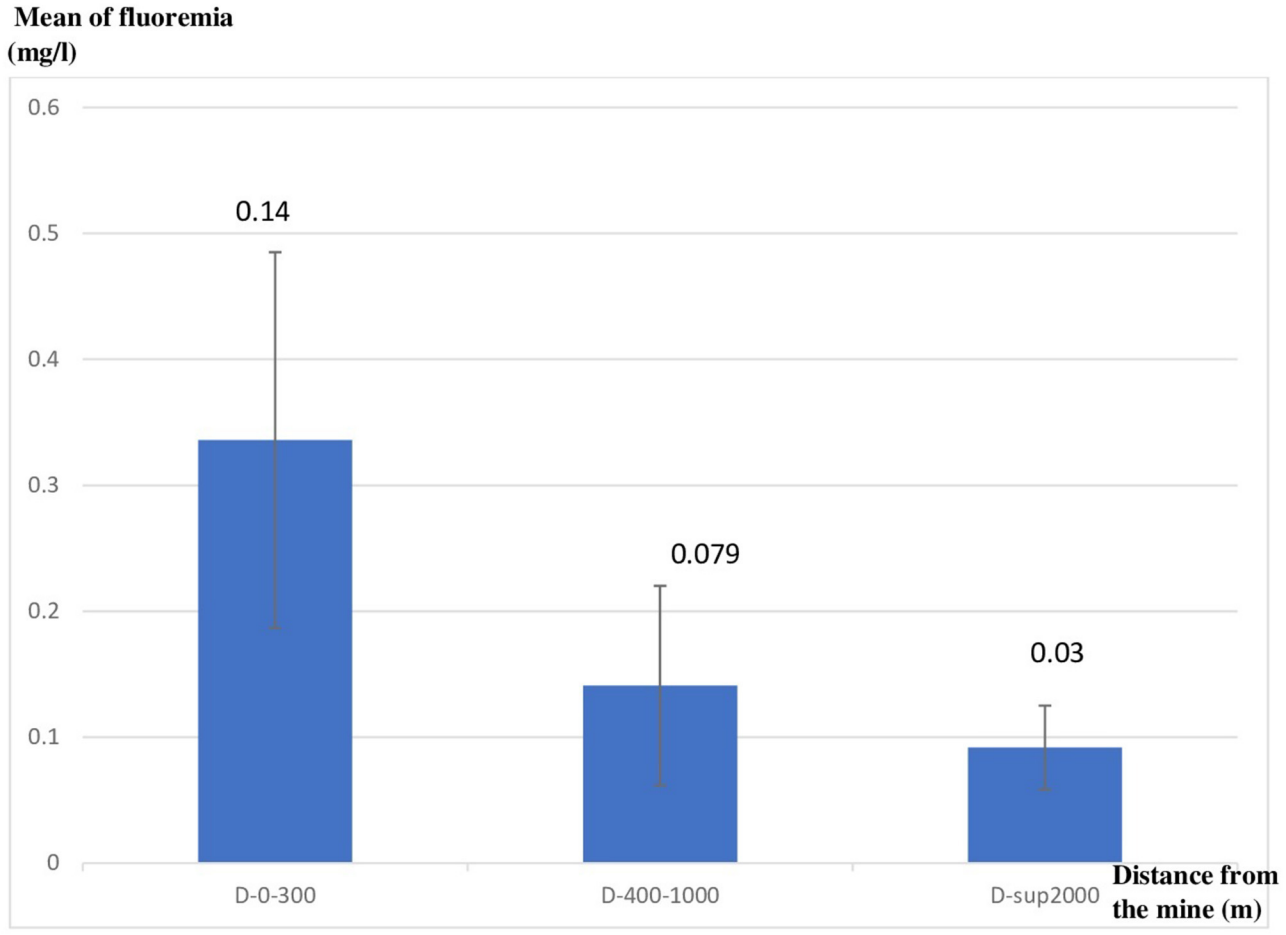
Mean of fluoremia by distance from the mine.

We also compared the mean of fluoremia levels of each group of animals based on their distance from the mine. The first group S1 had a mean fluoremia equal to 0.33 ± 0.14 mg/l, the second group S2 had a mean equal to 0.14 ± 0.07 mg/l and the third group S3 had a mean equal to 0.9 ± 0.03 mg/l. The difference was statistically significant (Figure 4).

Statistical analysis identified distance from mine as an independent risk factor for positive fluoremia with OR = 55; 95% confidence interval = [8.18–369.8] and *p* < 0.001.

The result of the correlation identified a Pearson coefficient *r* = −0.556 and *p* < 0.001.

## Discussion

In Tunisia, fluorosis is a real obstacle to the development of live stock farming in some regions of the country, particularly in southern Tunisia but also in some northern regions such as Hammam Zriba.

The Hammam Zriba region is a mining area for the extraction of fluoride and other minerals where fluoride toxicity could affect flora and fauna. Sheep are the most affected by fluorosis in this area. The majority of the farms are traditional, free-range farms. The animals drink water from the wadis as well as from surface water located near the abandoned fluoride mine.

Our results show that the fluoride levels in water samples collected near the mine are higher than those collected from a greater distance. Our results match those found by Bengoumi and Kessabi (2007), where the fluoride levels in water collected 200 m from a phosphate processing plant in southern Morocco are very high, in the order of 4.6 mg/l. This could be related to the weathering and erosion of the various mining discharges that seem to contribute to the contamination of the natural environment by fluoride.

Ambient fluorinated dust from abandoned mines and unconfined fluorite tailings containing a high fluoride load may be suspended by wind or transported by water runoff, or leached to ground water (Gonzalez-fernandez et al., 2011; Martinez et al., 2011).

High fluoride levels in ground water have been reported in other countries, particularly in England, France, Italy, and the USA. Fluoride concentrations range from 0.01 to 4 mg/1, but a predominance of values below 1 mg/l is still noted (Bertrand, 2001). These high fluoride concentrations recorded in deep waters can be related to the prolonged contact of alkaline water with fluoride-rich rocks, which leads to their release and passage through the water (Guissouma and Tarhouni, 2015). Sheep and cattle drinking from this water show dental and bone damage wich represented a major signs of fluorosis (Guissouma and Tarhouni, 2015).

As a consequence, they seem to be associated most often with ground water, with high pH values (>7) facilitating ion exchange between F- fluorides and OH- hydroxyl groups, and of the sodium bicarbonate type characterized by low concentrations of magnesium and calcium due to the low solubility of fluorine (Botha et al., 1993).

In our study, we noticed that high fluoride concentrations in water are related to the increase of its pH.

Similarly, the experimental results carried out by other study (Saxena and Ahmed, 2001) show that an alkaline environment (with a pH ranging from 7.6 to 8.6) is favorable to the dissolution and release of fluorine, which leads to high contents of this ore in the water analyzed.

Furthermore, in our study, we demonstrated that higher levels of fluoride in sheep drinking water are associated with higher levels of fluoride in the blood of animals drinking from these waters.

Our results demonstrate that the distance from the mine could be a determining factor in the contamination of animals by fluorine. Other researchers have also found the influence of distance from the site of fluorinated emissions on the level of fluorine in the blood of the animals examined (Djebbi et al., 2017).

A study carried out by Bengoumi and Kessabi in 2007, has shown an average fluoremia: 0.47 mg/l in camels living 200 m from a phosphate processing plant in southern Morocco.

In our study, we also showed that fluoremia increases with the age of the animals. This is explained by the fact that fluorine is a cumulative toxicant. After resorption, fluorides are rapidly transported through the blood stream throughout the body where they are stored in calcium-rich organs such as bones and teeth. Approximately 50% of the daily intake of fluoride is deposited in calcium-rich tissues and most of the fluoride in the body, 99%, is contained in bones and teeth, with the remainder distributed in the blood and richly vascularized organs (World Health Organization, 2002; Kaminsky et al., 2011). This process occurs in the short term, but especially in the long term where bones undergo bone remodeling (National Research Council, 2006). Therefore, fluoremia (determination of fluoride in the blood) is an excellent indicator of recent fluoride intake and a good indicator of environmental contamination. It should be determined periodically to determine the risk of fluorosis for at-risk herds.

Moreover, in young animals, 80–90% of the absorbed fluorides are retained in calcium-rich bones and teeth. This retention correlates with the resorbed dose per kilogram of body weight (b.w.); the higher the dose, the greater the retention (World Health Organization, 2002; Barbier et al., 2010). Therefore, the determination of fluorides is of no interest in young animals because of their high bone metabolism, especially in their ability to retain and accumulate fluoride in bones and teeth. Hence the plasma concentration of ionic fluoride is higher in older animals than in young animals (Milhaud et al., 1985; Zulfiya, 2011; Sezai and Ihsan, 2017).

Chronic exposure of animals to high levels of fluoride is of concern, hence the need for a prevention program to inform the public about the danger posed by fluoride present at high levels in the study area.

Prevention consists to encourage farmers to use drinking water for livestock animals and to watering crops, to keep food at a safe level by conducting periodic checks of water and animal food rations and to ensure a balanced diet.

## Conclusion

In this work we noted very high fluoride levels especially in the areas close to the mine. Therefore, the study area was highly contaminated by the fluoride for a 300 m radius from the mine (contaminated site) with a clear tendency to decrease while moving away from the study area.

## Data Availability Statement

The original contributions presented in the study are included in the article/supplementary material, further inquiries can be directed to the corresponding author/s.

## Ethics Statement

Ethical review and approval was not required for the animal study because Evaluation of toxicity in animal. Written informed consent was obtained from the owners for the participation of their animals in this study.

## Author Contributions

All authors listed have made a substantial, direct and intellectual contribution to the work, and approved it for publication.

## Conflict of Interest

The authors declare that the research was conducted in the absence of any commercial or financial relationships that could be construed as a potential conflict of interest.
